# MicroSTNet: a spatio-temporal graph-based framework for time-series microbiome analysis

**DOI:** 10.1099/mgen.0.001519

**Published:** 2025-10-03

**Authors:** Shichen Gao, Li Li, Jiajia Wang, Yadong Wang, Yan Dong

**Affiliations:** 1 College of Biology and Food Engineering, Chuzhou University, Chuzhou 239000, Anhui, PR China; 2 School of Life Sciences, Anhui University, Hefei 230601, Anhui, PR China; 3 Department of Neurosurgery, The Affiliated Chuzhou Department of Clinical Laboratory, Hospital of Anhui Medical University, The First People’s Hospital of Chuzhou, Chuzhou 239000, Anhui, PR China

**Keywords:** early disease diagnosis, interaction networks, microbial community structure, spatio-temporal dynamics

## Abstract

The structure and function of microbial communities are profoundly influenced by spatio-temporal dynamics. While existing machine learning algorithms are extensively used for phenotype prediction based on microbial communities, particularly for disease forecasting, they fail to fully utilize the spatio-temporal dynamics embedded in microbial data. Moreover, data collected at a single time point often proves inadequate for the accurate prediction of host or environmental phenotypes. This study investigates the interaction dynamics of microbial communities in closed environments using data from two independent research projects. We introduce the microbial spatio-temporal network model, which combines two-stream spatio-temporal graph convolutional networks with long short-term memory to predict dynamic microbial abundance in the human oral cavity and gut. The model captures the temporal trajectories of microbes together with spatial features embedded in network structures, enabling accurate prediction of future community trends. Experimental validation confirmed its ability to track temporal patterns with high accuracy, even for micro-organisms exhibiting significant fluctuations. Ablation experiments demonstrated that the integrated model outperforms individual components, harnessing the strengths of both approaches. This technology presents a promising strategy for low-cost, non-invasive early diagnosis of human diseases, offering valuable insights into future health risks.

**Impact Statement**

Although models for predicting phenotypes based on microbial abundance have been widely applied and are becoming increasingly effective, existing models fail to fully exploit the inherent spatio-temporal dynamics of microbial communities. Currently, no computational framework is available to process large-scale longitudinal datasets. We propose a microbial spatio-temporal network, a computational framework that accurately captures microbial temporal trajectories and the spatial features encoded within microbial abundance networks, while tracking the future abundance of microbial biomarkers across multiple time steps for various diseases. This framework offers a potential reference tool for low-cost, non-invasive early disease diagnosis and health status monitoring.

## Data Summary

We acquired the data from the “Moving Pictures of the Human Microbiome” project (MG-RAST:4457768.3-4459735.3) and the European Bioinformatics Institute European Nucleotide Archive (nucleotide accession number: ERP006059). The codes and datasets (including the complete operational taxonomic unit table, pretrained model weights, training data and test samples) are openly accessible on GitHub at https://github.com/pwner-web/MicroBioSTNet. All abundance matrix data used for model training and validation, comprising 1,595 samples from six cohorts, is accessible at https://github.com/pwner-web/MicroBioSTNet/tree/main/data/. The raw read data can be obtained from the European Bioinformatics Institute European Nucleotide Archive under the nucleotide accession number ERP006059. The computational code and weight files used in this study are available on GitHub under the MIT permissive open licence: https://github.com/pwner-web/MicroBioSTNet.

## Background

Microbial communities are inherently complex and dynamic, playing essential roles in various environments [1]. Their structure and function are shaped by spatial organization and temporal changes, which drive interactions that enhance community resilience and stability. These spatio-temporal dynamics can result in significant shifts in community behaviour and phenotypes over time [2,3]. Accurately capturing these patterns and predicting the future development of microbial communities is critical for anticipating host phenotypes.

In recent years, machine learning algorithms have become increasingly effective at leveraging microbial community data for phenotype prediction, particularly in disease assessment. The human gut microbiome has been extensively studied, revealing strong associations with various diseases and offering opportunities for novel therapeutic interventions [4]. For example, the gut microbiome has been implicated in diagnosing and potentially treating Alzheimer’s disease [5]. Ahmed A. Metally *et al*. demonstrated the potential of using longitudinal microbiome taxonomic profiles with long short-term memory (LSTM) networks to predict allergic responses based on abundance data [6]. Similarly, Sharma *et al*. developed TaxoNN, an ensemble neural network model applied to stratified microbiome data and successfully predicting diseases such as cirrhosis and type 2 diabetes, using operational taxonomic unit (OTU) abundances [7]. Standardized tools, such as SIAMCAT, created by Jakob Wirbel and colleagues, have further expanded the applications of gut microbiome-based models. SIAMCAT facilitates cross-disease comparisons and transferability across studies and has been applied to conditions including Crohn’s disease, colorectal cancer and type 2 diabetes [8].

The predictive potential of the oral microbiome has also received considerable attention. Salivary microbiome profiles, for instance, have been shown to predict the development of dental caries in teenagers over a 3-year period [9]. In addition, the salivary microbiome is being explored as a biomarker for chronic periodontitis progression [10]. Diversity and co-occurrence networks within the salivary microbiome have also been used to diagnose dental and periodontal health status, providing additional avenues for disease prediction based on OTU abundances [11].

Despite these advancements, there remains a dearth of large-scale longitudinal datasets and universal computational frameworks capable of handling them [12]. Existing models fall short in fully harnessing the temporal dynamics inherent in microbial community data. Models that depend exclusively on microbial data from a single time point prove insufficient for the accurate forecasting of future host phenotypes. Early detection and prediction of diseases are crucial for improving health outcomes and reducing healthcare costs. To address these limitations, we constructed interaction networks among taxa to explore the interaction characteristics of microbial communities in closed environments. Second, we developed a novel microbial abundance prediction model, microbial spatio-temporal network (MicroSTNet), by integrating two-stream spatio-temporal graph convolutional networks (2S-STGCN) with an LSTM model. Finally, we proposed a dynamic microbial prediction framework for microbial abundance in the human oral cavity and gut, offering a practical tool for low-cost, non-invasive early disease diagnosis.

## Methods

### Dataset description

We validated the performance of MicroSTNet using six real-world longitudinal datasets derived from 16S rRNA gene sequencing. These datasets comprised stool and saliva samples from three individuals, capturing the daily dynamics of gut and salivary microbial communities. Specifically, a total of 299 gut samples and 272 saliva samples were collected from subject A, 443 gut and saliva samples were collected from subject M3 over a 15-month period and 186 gut and saliva samples were collected from subject F4 over a 6-month period. All datasets were publicly accessible. Stool and salivary samples from subject A were collected by Lawrence A. David *et al*. [13], whereas those from subjects F4 and M3 were obtained by J. Gregory Caporaso *et al*. [14]. For this study, all six datasets were employed for both training and prediction and divided into training and validation sets in a 7:3 ratio.

### Sequence data analysis

**Fig. 1.**
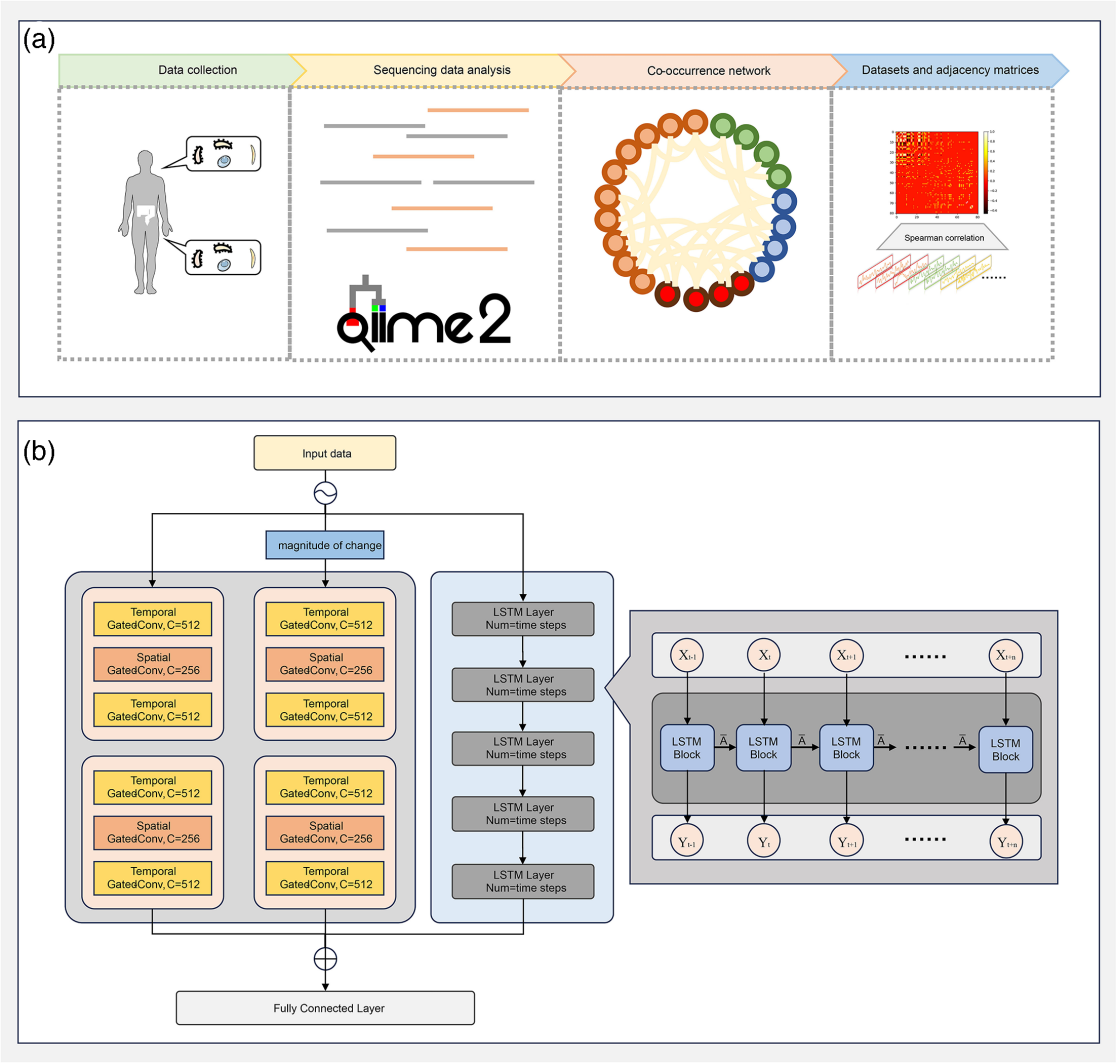
Overview of the workflow. The workflow was divided into two parts: (**a**) the upper section progresses sequentially from left to right. Initially, raw sequencing reads were collected from human saliva and stool samples. These reads were processed using QIIME2, involving re-replication, chimaera checking, OTU clustering and species annotation, to generate the abundance matrix. Taxon–taxon co-occurrence networks were then constructed using the Spearman method. Following this, the data were integrated into a time series for genus abundance alongside the adjacency matrix. (**b**) The lower section shows abundance changes between consecutive time steps from the abundance matrix, forming the ‘change stream.’ Both the time-based abundance stream (the abundance matrix) and the change stream, combined with the spatially informative co-occurrence networks matrix, were input into the model to predict abundance at the next time step.

We processed the raw reads using the QIIME2 analysis pipeline, developed by Evan Bolyen *et al*. (QIIME 2 version: 2021.11.0, hereafter referred to as q2) [15]. Initially, sequences were dereplicated with the q2-vsearch dereplicate-sequences method, and chimeric sequences were identified and removed using q2-vsearch uchime-denovo. The remaining sequences were clustered into OTUs through closed-reference clustering against the Greengenes 13_8 database at 99% identity using q2-vsearch cluster-features-closed-reference. Taxonomic assignments were performed with the q2-feature-classifier classify-sklearn method trained on the same reference database to ensure accurate annotation. The OTU table was subsequently collapsed to the genus level via the q2-taxa collapse method (level 6), and features with fewer than ten total counts across all samples or occurring in fewer than five samples were removed using q2-feature-table filter-features (Fig. 1).

### Data preprocessing

The datasets were then randomly divided into an 80% training set and a 20% test set. Several preprocessing steps were applied to both the training and test sets. Initially, the abundance of each OTU across multiple samples was calculated, and OTUs with zero abundance in all samples were removed. The data were then standardized using the *Z*-score method to normalize features across the datasets. Following these preprocessing steps, the final input data can be defined as follows:


$$X_{i,j,k}=\frac{X_{i,j,k}-\mu_{i}}{\sigma_{i}}$$

where the dataset *X* has three dimensions (𝑁, 𝐷, 𝑇), where *N* denotes the number of samples, *D* represents the feature dimension and *T* indicates the time steps. The mean array, means and the standard deviation array, Sd, have dimensions (*D*). Each element in the standardized dataset. The μi is the mean of the th feature, and σi is the standard deviation of the th feature.

The construction of the microbial co-occurrence network matrix is inspired by the method described by Lima-Mendez G *et al*. [16]. To begin, Spearman correlation coefficients and their corresponding *P*-values are calculated between each pair of datasets using the Spearman correlation method, as follows:


$$\rho =\frac{\frac{1}{n}\sum_{i=1}^{n}\left(R\left(x_{i}\right)-\bar{R\left(x\right)}\right)*\left(R\left(y_{i}\right)-\bar{R\left(y\right)}\right)}{\sqrt{\left(\frac{1}{n}\sum_{i=1}^{n}{\left(R\left(x_{i}\right)-\bar{R\left(x\right)}\right)}^{2}\right)*\left(\frac{1}{n}\sum_{i=1}^{n}{\left(R\left(y_{i}\right)-\bar{R\left(y\right)}\right)}^{2}\right)}}$$

$$\rho_{ij}=SpearmanRankCorrelationCoefficient\left(X_{i},X_{j}\right)$$

$$p_{ij}=pvalueusingpermutationtest(X_{i},X_{j})$$

*ρ* represents the Spearman rank correlation coefficient, measuring the strength and direction of the monotonic relationship between two variables. *R*(*x*) and *R*(*y*) denote the ranks of *x* and *y*, respectively, while $\bar{R(x)}$ and $\bar{R(y)}$ represent the mean ranks. *n* indicates the number of data points in each dataset. *p* refers to the *P*-value, which quantifies the probability of observing the given or more extreme results under the null hypothesis. *X* belongs to a set of real matrices with dimensions *X*∈*Rn*×*m*, where *n* represents the number of taxa (rows) and *m* the number of samples (columns). The Benjamini–Hochberg method is then applied to correct the *P*-values for multiple testing, resulting in adjusted *P*-values. Values in the Spearman matrix with *P*-values greater than 0.05 are subsequently removed.

### The MicroSTNet model

We proposed a fusion model based on a two-stream spatio-temporal graph convolutional network and LSTM. This model includes two input streams: the abundance stream and the change stream. Both streams share the same network architecture but operate on different input data (Fig. 1). The abundance stream uses microbial abundance at each time step as input, whereas the change stream utilizes the combined shifts in abundance between adjacent time steps as the input.

Network initialization is followed by spatio-temporal feature extraction using a combination of temporal and spatial graph convolutions. To improve the ability to capture long-range temporal dependencies, we used LSTM to extract temporal features from the abundance stream. For each stream, the features extracted from both the abundance and change streams were concatenated. The merged features were then passed through a fully connected layer, with the resulting output employed to predict the next time step or multiple future time steps.

### Temporal block

This module captures temporal features through convolutions along the time dimension. Each convolution operation computes the output as:


$$\;\text{conv}(X)[b,c,n,t]=\sum_{c_{\text{in}}=1}^{C_{\text{in}}}\sum_{i=1}^{1}\sum_{j=1}^{k}W[c,c_{\text{in}},i,j]\cdot X[b,c_{\text{in}},n,t-j+1]+b[c]$$

*W* represents the convolutional kernel weights. Bias terms adjust the output. The kernel size determines the window over which temporal features are extracted. By stacking multiple convolutions with linear and non-linear activations, the block captures both simple and complex temporal patterns. The final output, containing refined temporal features, is used in subsequent graph convolution operations.

### Spatial block

Let $T^{\left(l\right)}\in R^{N*T*C_{}}$ represent the input feature tensor at the lth layer, where *N* is the number of nodes, *T* is the number of timesteps, and *C* is the number of input channels. The normalized adjacency matrix Â$\in R^{N*N}$captures the spatial relationships between nodes.

The tensor lfs(l) is computed as follows:


$$lfs^{\left(l\right)}=\text{Einsum}(\hat{A},T^{l})$$

where Einsum represents the Einstein summation convention. Specifically, the operation aggregates the features of neighbouring nodes based on the structure of the graph:


$$\;lfS_{kilm}^{\left(l\right)}=\sum_{j=1}^{N}{\hat{A}}_{ij}*T_{jklm}^{\left(l\right)}$$

Thus, $lfs\in R^{B*N*T*C}$ captures the aggregated features for each node, influenced by its neighbours, where *B* is the batch size.

### LSTM block

**Fig. 2.**
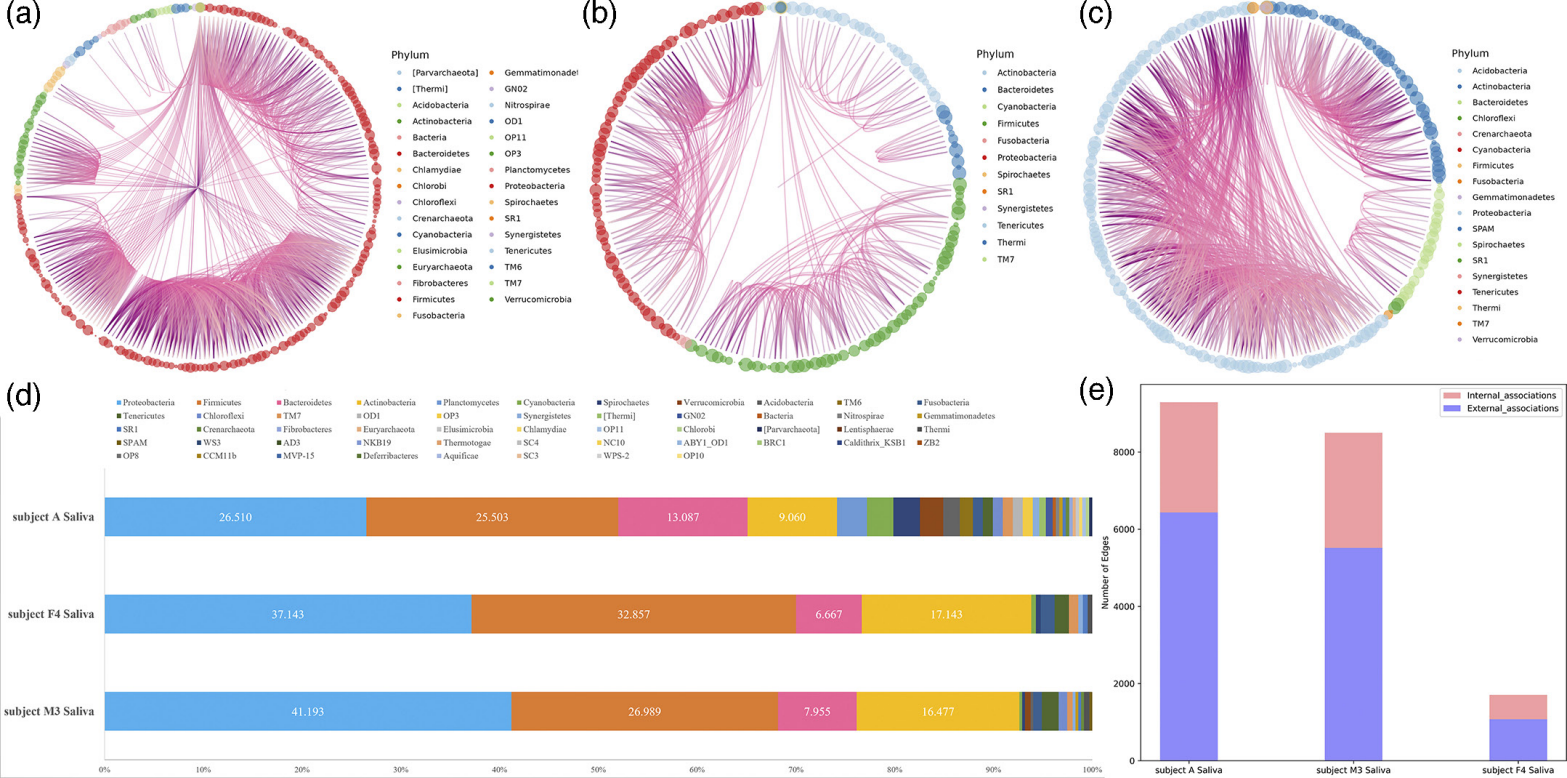
Performance evaluation of the three models. (a–h) present the MAE values of the models on the validation sets of six datasets as they evolve over epochs. In each subplot, the performance of the 2s-STGCN, MicroSTNet and LSTM models on the validation set is depicted by green, red and yellow lines, respectively.

We designed a module with a five-layer LSTM network seamlessly integrated into the 2s-STGCN framework (Fig. 1). Each LSTM layer contains units corresponding to the number of timesteps. Input data $X_{t}$ at each time step enter into the LSTM unit via the input gate, with information flow regulated by forget and output gates. The weights of these gates are represented by the transition matrix $\bar{A}$. Each LSTM unit incorporates three gating mechanisms (input, forget and output gates) and a memory cell state to manage information storage and propagation. Outputs from each LSTM layer feed into the subsequent layer, forming a deep network architecture. This design demonstrated exceptional performance, effectively capturing long-term dependencies (Fig. 2).

### Model training and evaluation

The running time for each model was measured using the built-in Windows time command. The hardware setup included an AMD Ryzen 7 8845 h CPU (16 logical processors, 3.80 GHz), an NVIDIA GeForce RTX 4070 Laptop GPU and 32 GB of RAM for both model training and prediction.

Model performance was assessed using the mean absolute error (MAE) and the symmetric mean absolute percentage error (sMAPE). The MAE was calculated as follows:


$$\;\text{MAE}=\frac{1}{m}\sum_{i=1}^{m}\left|y_{i}-{\hat{y}}_{i}\right|$$

where *m* is the number of samples, yi is the true value and $\hat{y_{i}}$ is the predicted value.

The sMAPE was computed as follows:


$$\text{sMAPE}=\frac{1}{m}\sum_{i=1}^{m}\frac{|y_{i}-{\hat{y}}_{i}|}{(|y_{i}|+|{\hat{y}}_{i}|)/2}\times 100\%$$

where *m* is the number of samples, yi is the true value and $\hat{y_{i}}$ is the predicted value.

## Results

### Construction of taxon–taxon co-occurrence networks

**Fig. 3.**
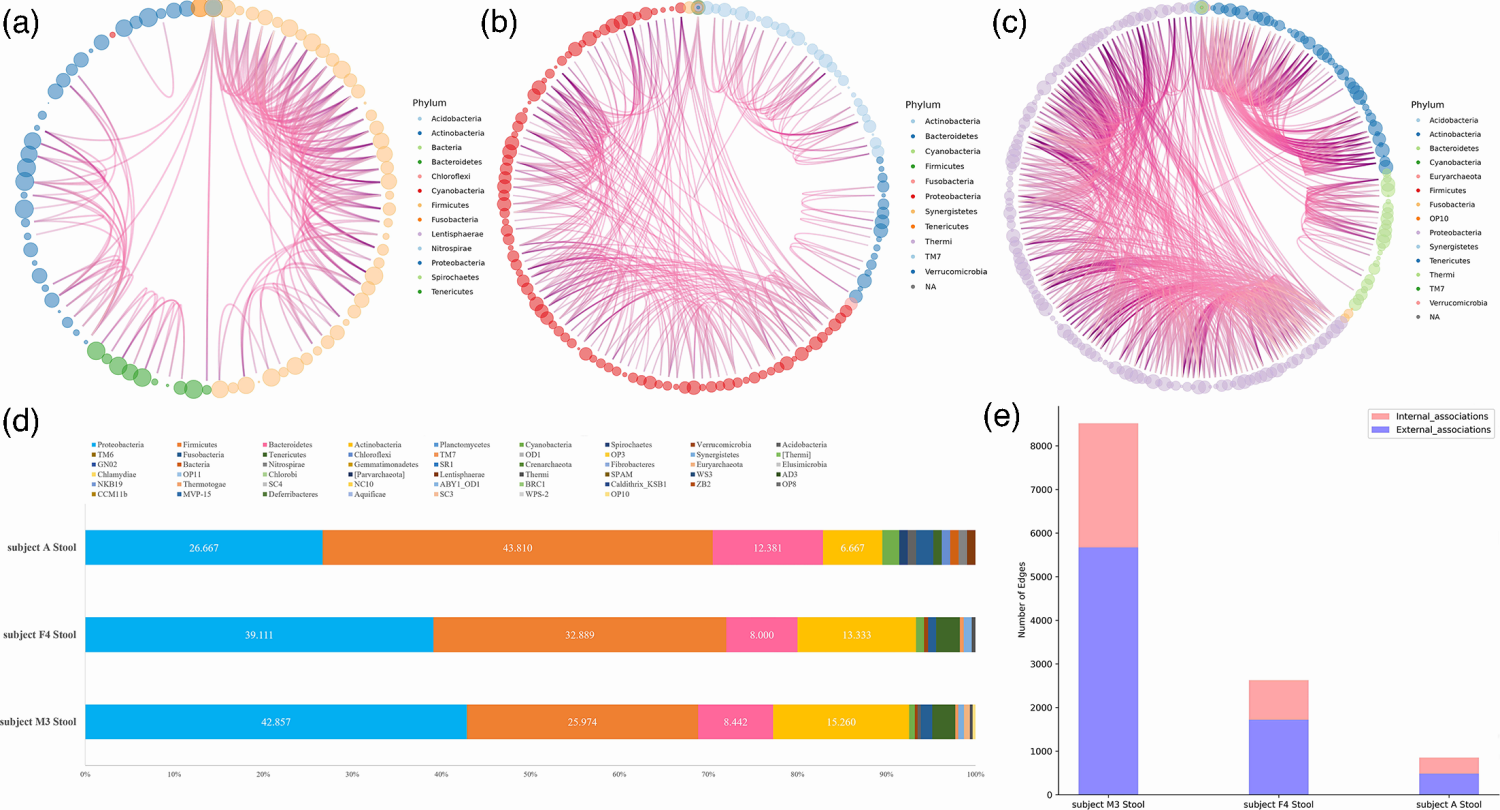
The three saliva samples’ taxonomic distribution within the associations network and the composition of each sample’s phylum. a–c Circos plots illustrating the distribution of association patterns in saliva samples from subject A (**a**), subject F4 (**b**) and subject M3 (**c**). The circles along the edges of the plots represent individual taxa, with taxa from the same phylum sharing the same colour. Thin bands connect OTUs, highlighting high-resolution interactions. (**d**) Distribution of OTUs at the phylum level in the saliva samples of subject A, subject F4 and subject M3 is shown, providing a comparative overview. (**e**) Distribution of internal associations (links within the same phylum) and external associations (links between different phyla) demonstrated that connectivity patterns among taxa were conserved at higher taxonomic levels.

Inspired by prior studies [16], we constructed taxon–taxon co-occurrence networks for six datasets (Fig. 1a). These networks comprised edges (predicted biotic interactions) and nodes (taxa at the genus level), with detailed methods outlined in the ‘Methods’ section [17,18]. Fig. 2a–c and 3a–c illustrate the co-occurrence networks for the six datasets, with Fig. 2a–c representing saliva samples and Fig. 3a–c depicting stool samples. In these figures, the size of the circles corresponds to the predicted number of interactions, and colours indicate groupings. Taxa from saliva and stool samples were grouped uniformly by phylum. Co-occurrence networks generated using the SparCC method are shown in Fig. S4.

**Fig. 4.**
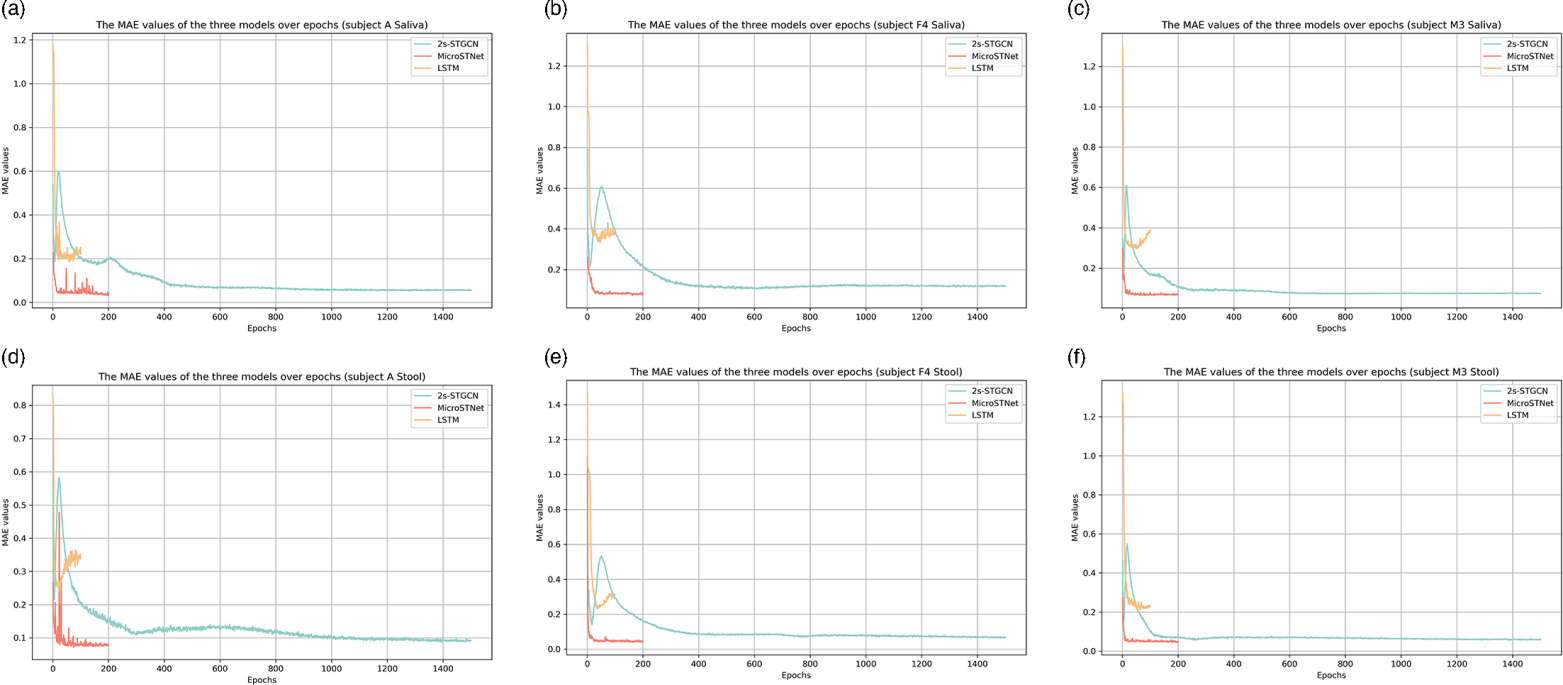
The three stool samples’ taxonomic distribution within the associations network and the composition of each sample’s phylum. a–c display Circos plots illustrating the distribution of association patterns in stool samples from subject A (**a**), subject F4 (**b**) and subject M3 (**c**). The circles at the outer edges represent various taxa, with those belonging to the same phylum sharing a common colour. High-resolution interactions between OTUs are depicted as thin connecting bands. (**d**) highlights the distribution of OTUs at the phylum level for subjects A, F4 and M3. (**e**) depicts the distribution of internal associations (links within the same phylum) and external associations (links between different phyla), demonstrating that connectivity patterns across taxa were conserved at higher taxonomic ranks within the gut microbiome.

The microbial samples collected from human hosts included saliva and stool; these samples were collected from subjects A, F4 and M3. Fig. 3 focuses on saliva samples, with Fig. 2a, e showing subject A’s sample exhibiting the highest microbial richness and network complexity, i.e. 9,293 edges (biotic interactions) and 299 nodes (taxa). Subject M3 had 8,517 edges and 309 nodes (Fig. 3c), while subject F4 had 1,707 edges and 211 nodes (Fig. 3b). In saliva samples, most edges stemmed from four phyla *– Proteobacteria*, *Firmicutes*, *Bacteroidetes* and *Actinobacteria –* which collectively accounted for over 50% of nodes (Fig. 3d). To assess alpha diversity, we calculated metrics such as Observed_features, Chao1, ACE, Shannon, Simpson, Pielou and Coverage across all samples for each subject (Figs S1, S2 and Table S1, available in the online Supplementary Material) [19,21]. In saliva samples (Fig. S1), differences in microbial richness and diversity were observed among the three subjects, with subject A generally showing higher values for richness and Shannon diversity compared to M3 and F4, while other metrics such as Simpson and Pielou showed more comparable levels across subjects.

Fig. 4 depicts stool samples; Fig. 3c, e reveals that subject M3’s stool sample exhibited the most edges (8,517) and the highest number of nodes (309). Subject F4 had 2,625 edges and 226 nodes (Fig. 3b, e), consistent with patterns observed in saliva samples. In contrast, subject A had 853 edges and 106 nodes (Fig. 3a, e), opposite to the co-occurrence patterns seen in saliva, which may reflect individual variation in gut microbial networks, as previously observed under certain conditions [22]. As in the saliva samples, the majority of edges in these networks were attributed to the phyla *Proteobacteria*, *Firmicutes*, *Bacteroidetes* and *Actinobacteria*, which collectively constituted over 50% of the nodes (Fig. 4d).

Microbial richness varied significantly across subjects. Nonetheless, co-occurrence patterns were consistent, with all samples showing a predominance of external associations. This suggested that the ecological structures of the oral and gut microbiomes shared similar co-occurrence modes across all three subjects (Figs3e  4e).

### Model performance evaluation

Mean Absolute Error (MAE) is a more intuitive metric for average error, being more robust to outliers than Mean Squared Error (MSE) and offering greater clarity than Root Mean Squared Error (RMSE) [18]. Consequently, evaluations and comparisons of average model performance were based on MAE. For the three models – MicroSTNet, LSTM and 2s-STGCN – we selected the model weights with the smallest MAE from each dataset to make predictions on their respective validation sets. Our MicroSTNet model performed exceptionally well across all six datasets. Fig. 2 presents the MAE values for each dataset. The model exhibited rapid convergence across datasets of varying structural complexity, time steps and time spans, achieving convergence in fewer than 50 epochs in all cases. Furthermore, it consistently achieved the lowest MAE values across datasets (Table 1). Although the standalone LSTM also converged quickly, its MAE values were higher. Ablation experiments revealed that removing the LSTM from the 2s-STGCN hindered convergence and increased MAE values (Table 1), highlighting the efficacy and broad generalizability of our model.

**Table 1.** Comparison of MAE values for three models – MicroSTNet, LSTM and 2s-STGCN – used to predict datasets from different subjects

Dataset	Algorithm	MAE value	Computing time per run (min)
Subject A saliva	MicroSTNet	**0.0325**	56
	2s-STGCN	0.0535	312
	LSTM	0.1882	42
Subject A stool	MicroSTNet	**0.0723**	39
	2s-STGCN	0.0878	154
	LSTM	0.2392	10
Subject F4 saliva	MicroSTNet	**0.0736**	18
	2s-STGCN	0.1047	95
	LSTM	0.3337	6
Subject F4 stool	MicroSTNet	**0.0391**	19
	2s-STGCN	0.0633	107
	LSTM	0.2329	5
Subject M3 saliva	MicroSTNet	**0.0653**	50
	2s-STGCN	0.0726	699
	LSTM	0.2966	23
Subject M3 stool	MicroSTNet	**0.0445**	31
	2s-STGCN	0.0562	312
	LSTM	0.2116	19

Boldface indicates instances where the MicroSTNet model significantly outperformed the other methods in terms of prediction accuracy.

Interestingly, model performance and microbial richness in the datasets appeared to vary in tandem. In the saliva dataset, subject A exhibited high species richness, with over 290 nodes and 9,000 edges (Fig. 2a, e). Conversely, in the stool dataset, subject A had the lowest richness, with only 106 nodes and 853 edges (Fig. 3a, e). Notably, subject A’s saliva dataset achieved the lowest MAE among all saliva datasets, while its stool dataset exhibited the highest MAE among stool datasets (Table 1). These observations are consistent with patterns of microbial richness and co-occurrence, suggesting a possible association with training outcomes. However, this tendency was not observed in the LSTM model, where MAE values remained consistently around 0.2 across datasets (Table 1). This indicates that MicroSTNet and 2s-STGCN are particularly effective at capturing spatial features. The superior performance of MicroSTNet across all datasets likely stems from its integration of the spatial feature capture capabilities of graph neural networks with the enhanced temporal feature extraction provided by the LSTM module.

### Prediction results from human saliva sample data

**Fig. 5.**
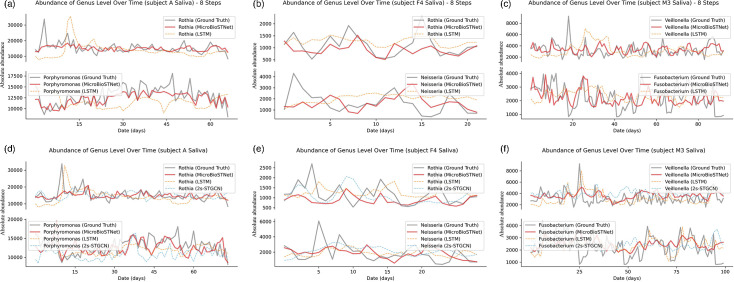
Comparative analysis of the three models’ predicted values versus actual values at the genus level in the validation datasets of saliva samples from subjects A, F4 and M3. (a–c) compare the predicted values for the next eight steps, with predictions from the MicroSTNet model shown as solid red lines, those from the LSTM model as dashed yellow lines and the actual values as solid grey lines for the validation sets of three datasets. The lower section (d–f) presents the predicted values of the three models MicroSTNet (solid red lines), LSTM (dashed yellow lines) and 2s-STGCN (dashed blue lines), alongside the actual values (solid grey lines).

Figs.5  6, along with Fig. S3, illustrate the predictive performance of the model on salivary microbiome datasets, evaluated at both the genus and phylum levels.

**Fig. 6.**
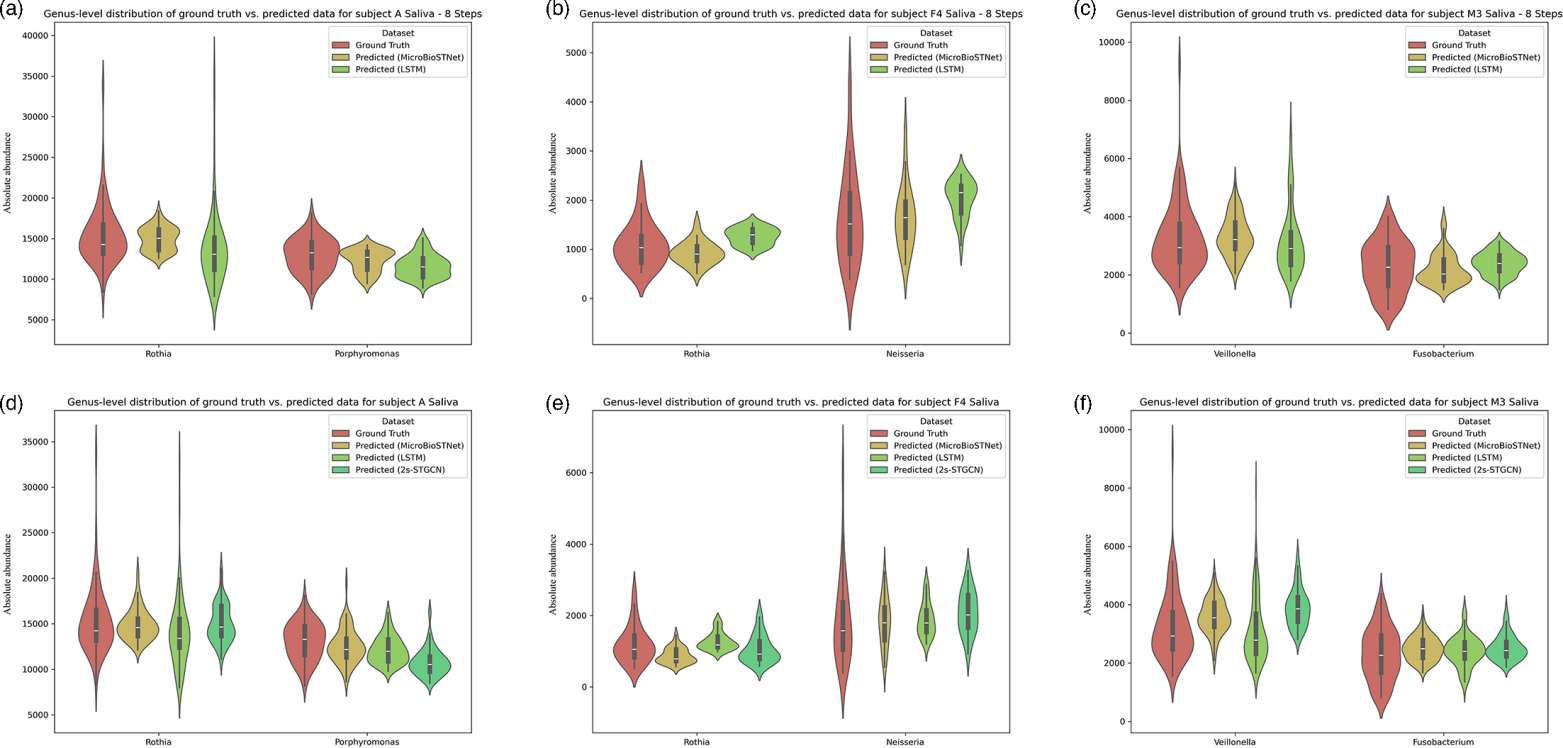
Distribution of the model’s predicted values versus actual values at the genus level in the validation datasets of saliva samples from subjects A, F4 and M3. The upper section (a–c) displays predictions for the next eight steps, whereas the lower section (d–f) focuses on predictions for the next single step. Violin plots were used to visualize the distribution of predicted versus actual values, showcasing the median, interquartile range and overall data distribution. These plots provide a statistical comparison between the model predictions and actual values. The distribution of actual values is represented in red, whereas the predictions from the MicroSTNet, LSTM and 2s-STGCN models are shown in yellow, light green and dark green, respectively.

In Fig. 5, the upper and lower sections depict the model’s performance for one-step-ahead and multi-step-ahead (eight steps) predictions, respectively. Subplots a, b and c display temporal changes in the abundance of various genera over the next eight time steps for subjects A, F4 and M3. Subplots d, e and f illustrate one-step-ahead predictions for the same subjects. The grey solid lines represent actual values, red solid lines show predictions by MicroSTNet, orange dashed lines correspond to LSTM predictions and blue dashed lines represent predictions by the 2s-STGCN model. Two genera with the most significant fluctuations were analysed in detail. As shown in Fig. 5a, d, the model accurately predicted the abundance of the genus *Rothia* and *Porphyromonas*, capturing short-term variations and overall trends effectively. However, LSTM predictions exhibited a noticeable lag. In Fig. 5b, e, predictions for the genera *Neisseria* and *Rothia* closely aligned with actual values, with all models effectively capturing their fluctuations and abundance changes. In Fig. 5c, predictions for the genera *Veillonella* and *Fusobacterium* nearly overlapped with the actual data; however, LSTM showed a slight lag in one-step-ahead predictions, while 2s-STGCN predictions displayed minor outliers. In the multi-step-ahead predictions (eight steps), LSTM results included spurious peaks, whereas MicroSTNet maintained greater accuracy and consistency.

Fig. 6 presents violin plots corresponding to Fig. 5, illustrating the distribution of actual and predicted values for the saliva dataset. In most cases, the predicted values from MicroSTNet exhibit distributions closest to the actual values. Notably, in Fig. 6a, d for the genus *Rothia* and Fig. 6c, f for the genus *Veillonella*, LSTM predictions appear visually closer to actual values than MicroSTNet’s. However, as highlighted in Fig. 5, LSTM predictions lag while attempting to fit anomalous peaks, compromising their accuracy and real-time responsiveness.

Fig. S3 focuses on the four major phyla predominant in the dataset – *Proteobacteria*, *Firmicutes*, *Bacteroidetes* and *Actinobacteria* – to compare temporal changes in abundance. The upper section (subplots a, b and c) shows these changes in the saliva of subjects A, F4 and M3. The grey solid lines denote actual values, red solid lines indicate MicroSTNet predictions, orange dashed lines show LSTM predictions and blue dashed lines represent 2s-STGCN predictions. Coloured shaded regions correspond to 95% confidence intervals for each model. Predictions from all three models align closely with actual data, demonstrating minimal deviation and high prediction confidence; detailed performance evaluation data are provided in Table S2.

### Prediction results on the human stool sample data

**Fig. 7.**
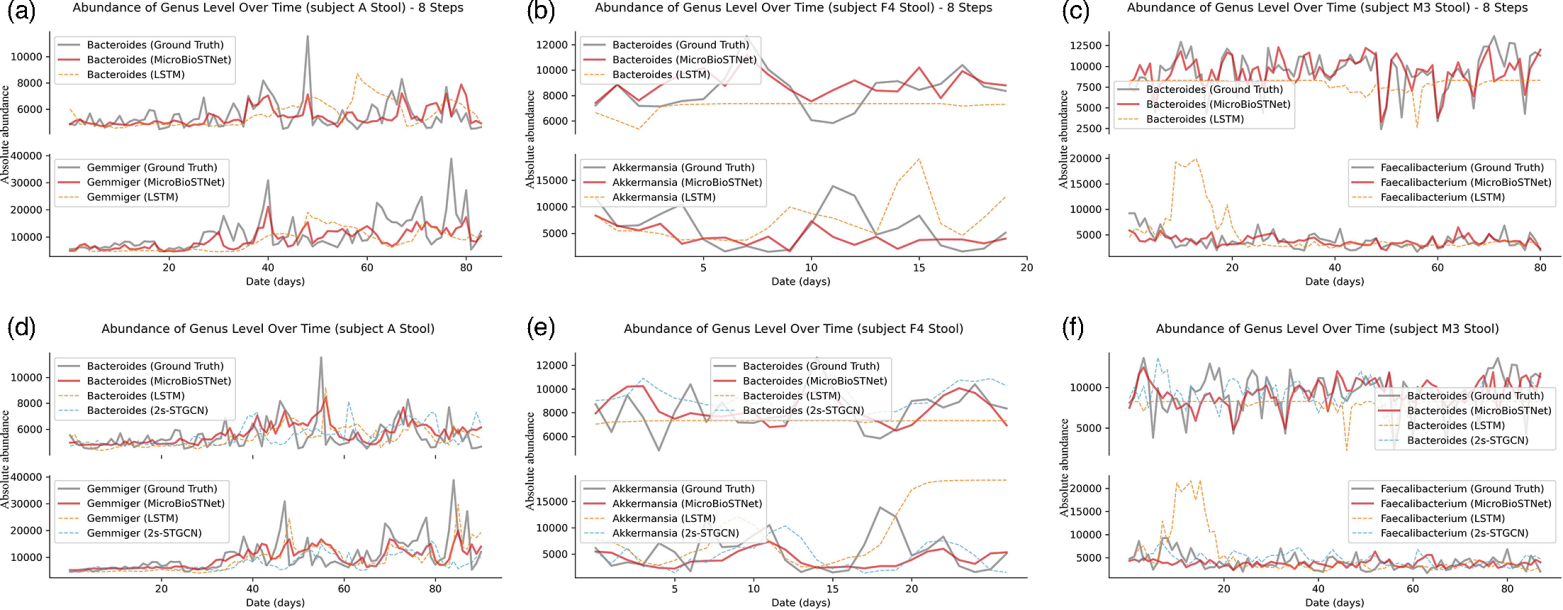
Comparative analysis of the three models’ predicted values vs. actual values at the genus level in the validation datasets of stool samples from subjects A, F4 and M3. (a–c) compare the predicted values for the next eight steps, with predictions from the MicroSTNet model shown as solid red lines, those from the LSTM model as dashed yellow lines and the actual values as solid grey lines for the validation sets of the three datasets. The lower section (d–f) presents the predicted values from the three models – MicroSTNet (solid red lines), LSTM (dashed yellow lines) and 2s-STGCN (dashed blue lines) – alongside the actual values (solid grey lines).

**Fig. 8.**
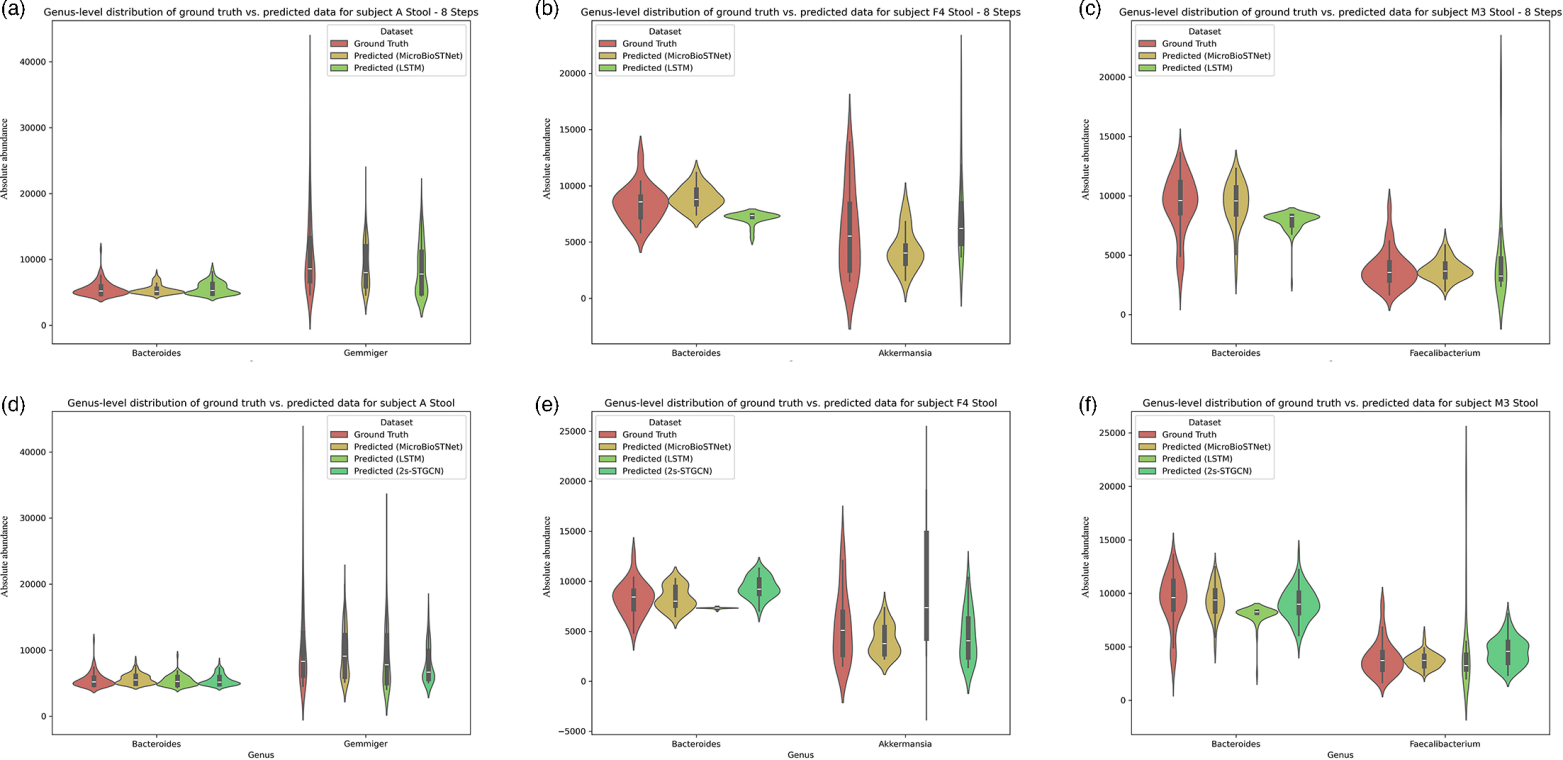
Distribution of the model’s predicted values vs. actual values at the genus level in the validation datasets of stool samples from subjects A, F4 and M3. The upper section (a–c) illustrates predictions for the next eight steps, whereas the lower section (d–f) focuses on predictions for the next single step. Violin plots are used to depict the distribution of predicted versus actual values, highlighting the median, interquartile range and overall data distribution. These plots provide a statistical comparison between the model predictions and the actual values. The distribution of actual values is represented in red, whereas the predictions from the MicroSTNet, LSTM and 2s-STGCN models are shown in yellow, light green and dark green, respectively.

Fig. 7 highlights the model’s performance in one-step and multi-step predictions of microbial time series at the genus level across the three stool datasets. Similar to the analysis for saliva datasets, the two genera with the greatest fluctuations were selected for demonstration. In Fig. 7d, the genera *Bacteroides* and *Gemmiger* in subject A’s dataset show robust one-step prediction results. Despite their pronounced fluctuations, all three models effectively track trends, with predicted values aligning closely with actual data. However, in eight-step predictions, LSTM struggles, producing spurious peaks (Fig. 7a). Corresponding violin plots (Fig. 8a, d) show similar distributions of predicted and actual values across the models, although MicroSTNet maintains the closest alignment overall. At the phylum level for subject A’s stool dataset, all models performed well on the phyla *Firmicutes* and *Bacteroidetes*, but LSTM predictions for the phyla *Proteobacteria* and *Actinobacteria* showed greater deviation from actual values, failing to capture occasional small peaks (Fig. S3d).

For subject F4, as shown in Fig. 7e, LSTM demonstrates significant shortcomings in short-term predictions. For instance, in one-step predictions, forecasted values for the genus *Akkermansia* deviate significantly from actual data toward the end, while the genus *Bacteroides* predictions appear unnaturally flat. In contrast, MicroSTNet and 2s-STGCN capture peak and trough variations accurately, with predictions nearly coinciding with actual values, effectively reflecting both fluctuations and overall trends. This precision extends to eight-step predictions (Fig. 7b), where MicroSTNet continues to outperform. Violin plots (Fig. 8b, e) reinforce this observation, showing MicroSTNet’s predicted values as closest to the actual data distribution, while LSTM diverges significantly. At the phylum level for subject F4’s stool dataset, MicroSTNet maintains superior performance. While all three models perform well for the phylum *Bacteroidetes*, both LSTM and 2s-STGCN lag behind MicroSTNet in predicting the phyla *Firmicutes*, *Proteobacteria* and *Actinobacteria* (Fig. S3e).

LSTM also performed poorly on subject M3’s dataset, producing numerous hallucinations and errors in one-step and eight-step predictions for the genera *Bacteroides* and *Faecalibacterium*. In contrast, predictions by MicroSTNet and 2s-STGCN demonstrated a high level of concordance with the actual data, with MicroSTNet excelling due to its ability to integrate spatial and temporal feature capture, resulting in superior prediction accuracy (Fig. 7c, f). As shown in Fig. 8c, f, MicroSTNet’s predicted values consistently aligned closely with actual distributions, whereas LSTM’s predictions failed to match the actual data, showing significant discrepancies. At the phylum level for subject M3’s dataset (Fig. S3f), the three models showed comparable performance overall. However, LSTM displayed slight anomalies, including lower-than-expected values during the middle and end of predictions for the phylum *Actinobacteria*; detailed performance evaluation data are provided in Supplementary Table S2.

## Discussion

Traditional time series algorithms face significant limitations in handling multivariate time series, making the prediction and modelling of human microbiome time series a persistent challenge [23]. A primary issue with multivariate time series forecasting is the interdependence of variables. However, traditional time series models like LSTM fail to fully exploit the spatial dependencies between variable pairs, resulting in distorted predictions and the appearance of false peaks or troughs (Figs5a  7c, f) [23]. Conversely, graph neural networks (GNNs) utilize a well-defined graph structure for information propagation, enabling them to effectively capture these interdependencies. The MicroSTNet model we developed integrates the topological feature capture capabilities of GNNs with the temporal pattern extraction strengths of LSTM. This combination has demonstrated remarkable efficacy, producing the best outcomes in both ablation and comparison experiments.

The study of microbial abundance at the phylum level within the human body revealed that the gastrointestinal microbiota of an adult typically comprises 500 to 1,000 distinct bacterial species, predominantly from the following four phyla: *Firmicutes*, *Bacteroides*, *Proteobacteria* and *Actinobacteria* [24,25]. Our findings aligned with this observation, as similar patterns were observed for the four phyla – *Firmicutes*, *Bacteroides*, *Proteobacteria* and *Actinobacteria –* in saliva and stool samples from all three subjects (Figs3d  4d).

Saliva samples showed that the genera *Rothia*, *Porphyromonas*, *Neisseria*, *Veillonella* and *Fusobacterium* exhibited notable fluctuations across the three datasets. Among these, the genus *Rothia* appeared frequently in both subjects A and F4, suggesting a potential pattern worth further investigation. These genera are frequently employed in machine learning and deep learning models across various domains. *Rothia*, a genus of pleomorphic Gram-positive bacteria present in the human oral, intestinal and skin microbiota, is recognized as an opportunistic pathogen. It plays a critical role in machine learning models for predicting dental caries. Research by Steve R. Gill *et al*. demonstrated that the genus *Rothia* increases caries risk, establishing its significance as a biomarker [26]. Similarly, Yan Wei Lim *et al.* identified *Rothia* as prevalent in the sputum of individuals with cystic fibrosis [27].

Furthermore, the genera *Rothia* and *Neisseria*, which are linked to periodontal health, have been shown to inversely correlate with inflammatory cytokines [28,30]. Their increased presence in nitrate-rich environments may play a role in reducing inflammation [31]. *Neisseria*, typically enriched in the oral cavities of healthy individuals [32,33], is a key feature in machine learning models used to classify caries groups [34]. A decrease in its abundance in oral cancer samples could make it a potential diagnostic marker, improving the precision of these models [35].

In addition, Jialin Lyu’s machine learning model for predicting Coronavirus Disease 2019 (COVID-19) severity demonstrated strong performance metrics (accuracy 0.82, F1-score 0.88, AUC 0.83), identifying the genera *Neisseria*, *Veillonella* and *Rothia* as critical biomarkers driving its accuracy. These microbes exhibit a significant association with COVID-19 severity [36]. Furthermore, a study by Xiao Zeng *et al.* on persistent pulmonary nodules utilized a machine learning model to identify the genera *Fusobacterium* and *Porphyromonas* as essential biomarkers for distinguishing patients with these nodules from healthy individuals. This research also highlighted the altered immune responses and metabolic activities of the salivary microbiome in affected patients [37].

In stool samples, the genera *Bacteroides*, *Gemmiger*, *Akkermansia* and *Faecalibacterium* also demonstrated apparent fluctuations across the three datasets, with the genus *Bacteroides* consistently observed to vary across all datasets. The genus *Bacteroides* is integral to predictive models in machine learning and deep learning. Research indicates that *Bacteroides uniformis* is strongly linked to a healthy microbiome and serves as a classification marker in machine learning [38]. Conversely, *Bacteroides dorei*, *Bacteroides eggerthii* and *Bacteroides thetaiotaomicron* are identified as obesity biomarkers, with their modulation potentially aiding in the mitigation of obesity and related complications [39,42]. The decline of these species in diabetic patients highlights their critical role in disease progression [43,44]. A previous study on type 1 diabetes revealed that various *Bacteroides* species dominate model predictions, emphasizing their importance for machine learning performance [45]. Additionally, Xuangao Wu *et al*. confirmed that the genus *Bacteroides* significantly enhances predictive outcomes in ulcerative colitis models through SHapley Additive exPlanations analysis [46]. The genus *Faecalibacterium* is recognized as a potential driver in colorectal cancer [47] and a key component of a healthy microbiome [48,49]. It is positively associated with moderate or healthy states in irritable bowel syndrome [50]. The genus *Akkermansia* has been observed in higher abundance among patients with colorectal cancer (CRC) compared with controls, effectively distinguishing patients with CRC in machine learning models. As a biomarker, it achieved an area under the receiver operating characteristic curve value of 0.925 in a validation cohort, underscoring its potential as a CRC biomarker [51]. In a study by Qi Wang *et al*., using machine learning to predict primary biliary cholangitis (PBC) progression to cirrhosis, the genus *Gemmiger* was identified as the predominant gut microbiota in PBC patients with cirrhosis, serving as a non-invasive biomarker (AUC=0.902) [52]. The abundance of the genus *Gemmiger* also shows promise as a non-invasive biomarker for distinguishing patients with Crohn’s disease [53].

Our model effectively captured temporal variations in the abundance of these key species across the aforementioned human microbiome datasets, offering a reliable, low-cost and non-invasive tool for the early diagnosis of potential diseases.

## Conclusion

We herein developed a microbial abundance prediction model with genus-level precision, adept at capturing the temporal dynamics of microbes in relatively closed environments, such as the oral cavity and gut. This capability enables precise predictions of future structural features and trends within these ecosystems, a conclusion validated through experimental results.

Our findings revealed that the developed model accurately tracked nearly all microbes with significant fluctuations in terms of their temporal patterns, enabling highly precise predictions of their future behaviour. Notably, these microbes, characterized by pronounced variability, have also been identified as key variables in other studies utilizing machine learning or deep learning to predict phenotypes based on microbial abundance. This highlights their essential role in enhancing model accuracy and suggests the potential to develop a robust pipeline integrating these models for phenotype prediction through forecasting future microbial abundance. This technology holds promise for low-cost, non-invasive early diagnosis of human diseases, providing valuable insights into potential health risks.

While highly effective machine learning and deep learning models are widely used for phenotype identification with microbial sequencing data, their applicability often requires retraining when applied to different studies or diseases, thereby limiting their direct transferability. This challenge highlights the complexity of creating a reliable pipeline for early disease diagnosis. To address this, future efforts should focus on developing transfer learning techniques that leverage existing models, enabling the creation of more versatile and adaptable diagnostic tools.

## Supplementary material

10.1099/mgen.0.001519 Uncited Supplementary Material 1.

10.1099/mgen.0.001519 Uncited Supplementary Material 2.

10.1099/mgen.0.001519 Uncited Supplementary Material 3.

## Abbreviations

CRC: colorectal cancer
GNNs: graph neural networks
LSTM: long short-term memory
MAE: mean absolute error
MicroSTNet: microbial spatio-temporal network
MSE: Mean Squared Error
OTU: operational taxonomic unit
PBC: primary biliary cholangitis
RMSE: Root Mean Squared Error
sMAPE: symmetric mean absolute percentage error
2s-STGCN: spatio-temporal graph convolutional networks
