# Supplementary material for: MicroSTNet: a spatio-temporal graph-based framework for time-series microbiome analysis
Source: Microb Genom. 2025 Oct 3;11(10):001519. doi: 10.1099/mgen.0.001519 (PMC12546993; doi:10.1099/mgen.0.001519)
Supplement: Uncited Supplementary Material 1. [file mgen-11-01519-s001.pdf]

Supplementary Figures

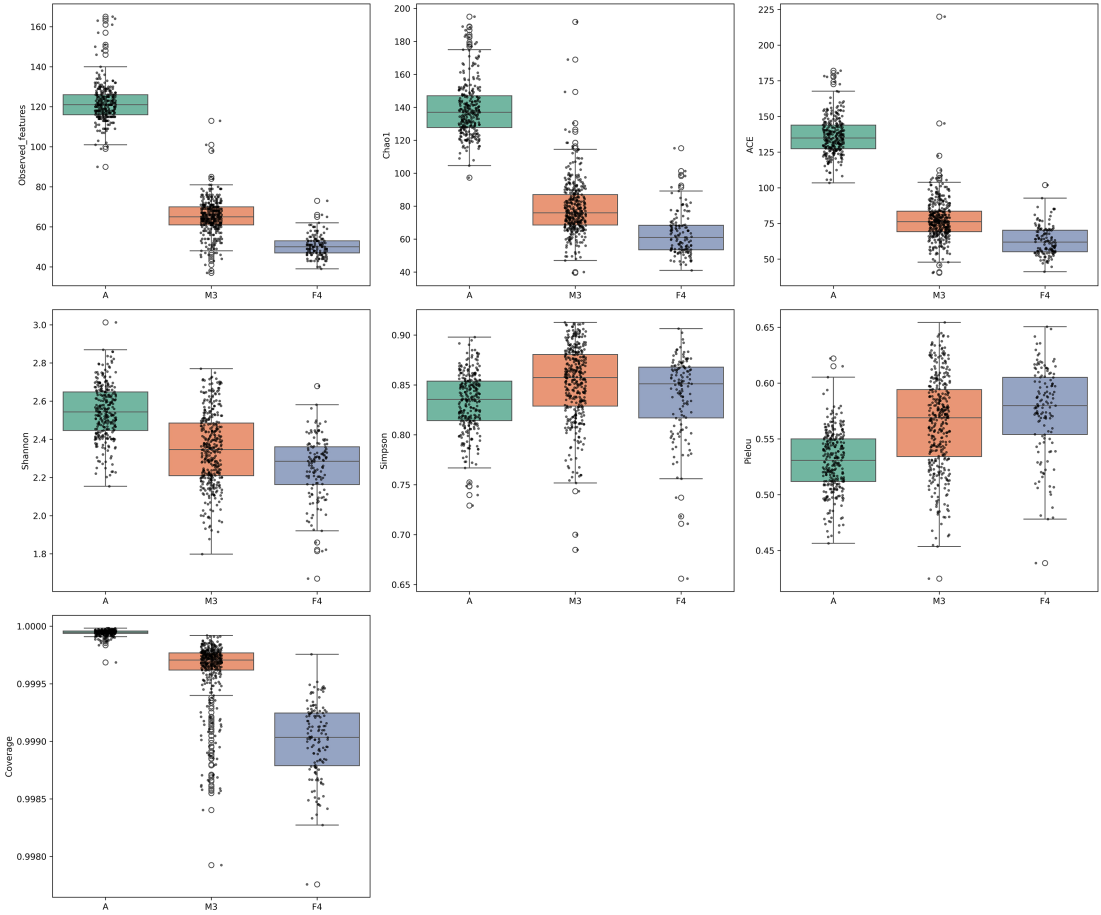

Supplementary Figure 1 Alpha diversity analysis across saliva samples from different subject groups (A, M3, F4).

This figure displays the alpha diversity metrics for saliva samples from three distinct subject groups, labeled A, M3, and F4. The box plots depict the distribution of various alpha diversity indices, including Observed Features, Chao1, ACE, Shannon, Simpson, Pielou, and Coverage. Each box plot shows the median value (horizontal line within the box), the interquartile range (box height), and outliers (points beyond the whiskers). The Observed Features index indicates the total number of unique taxa detected in each group. Chao1 and ACE are non-parametric estimators used to infer species richness.

Shannon and Simpson indices quantify diversity by considering both species richness and evenness. Pielou’s evenness index measures how evenly individuals are distributed among the species present. Coverage reflects the completeness of sampling.

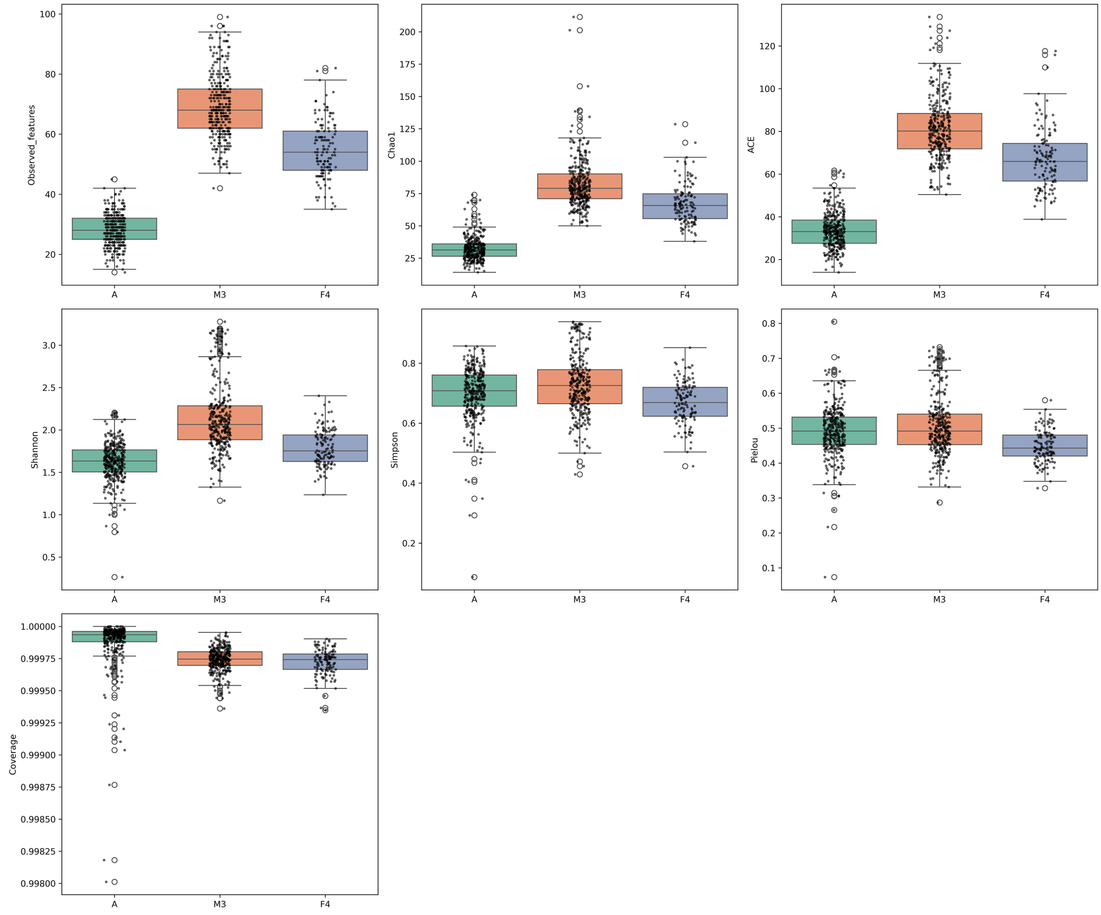

Supplementary Figure 2 Alpha diversity analysis across stool samples from different subject groups (A, M3, F4).

This figure displays the alpha diversity metrics for stool samples from three distinct subject groups, labeled A, M3, and F4. The box plots depict the distribution of various alpha diversity indices, including Observed Features, Chao1, ACE, Shannon, Simpson, Pielou, and Coverage. Each box plot shows the median value (horizontal line within the box), the interquartile range (box height), and outliers (points beyond the whiskers).

The Observed Features index indicates the total number of unique taxa detected in each group. Chao1 and ACE are non-parametric estimators used to infer species richness. Shannon and Simpson indices quantify diversity by considering both species richness and evenness. Pielou’s evenness index measures how evenly individuals are distributed among the species present. Coverage reflects the completeness of sampling.

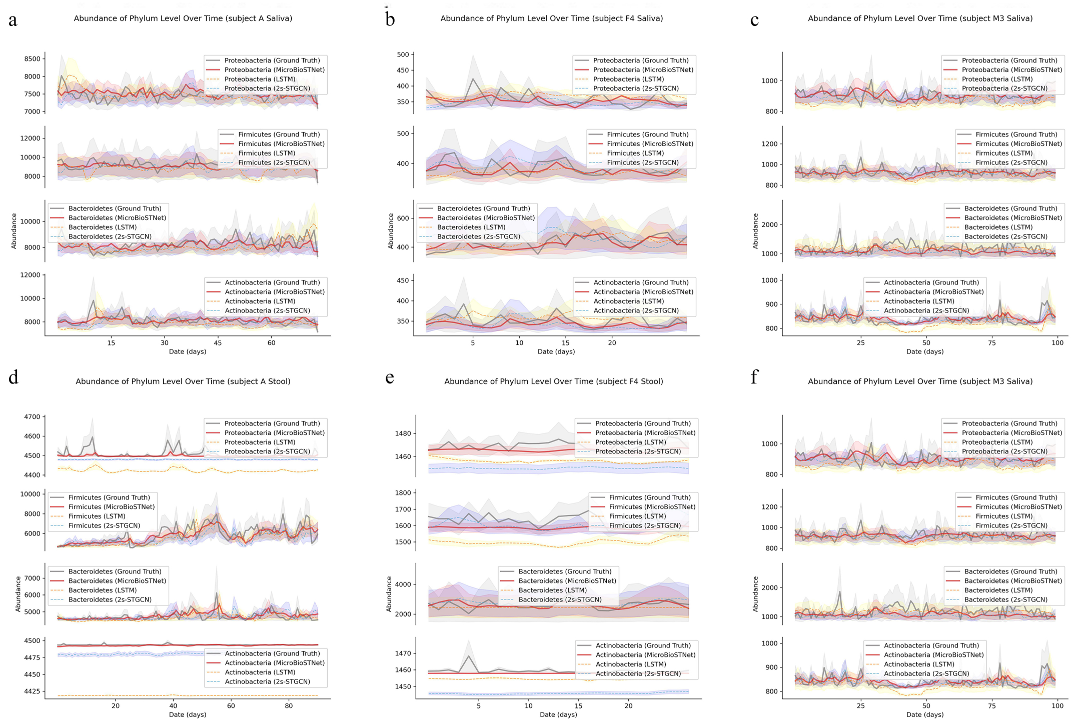

Supplementary Figure 3 A comprehensive comparative analysis of the model’s average predicted values versus average actual values at the phylum level in the validation datasets of saliva and stool samples from subjects A, F4, and M3.

The prediction performance of the model at the phylum level is shown for both saliva samples (a–c) and stool samples (d–f). In each subplot, predictions from the three models are represented as red solid lines for the Microbial Spatio-Temporal Network (MicroSTNet) model, yellow dashed lines for the Long Short-Term Memory (LSTM)

model, and blue dashed lines for the 2s-STGCN model, whereas the actual values are depicted as solid gray lines. Each subplot also includes the mean and 95% confidence interval (shaded area around the curves) for different phyla, enabling a clear comparison of the models' performance.

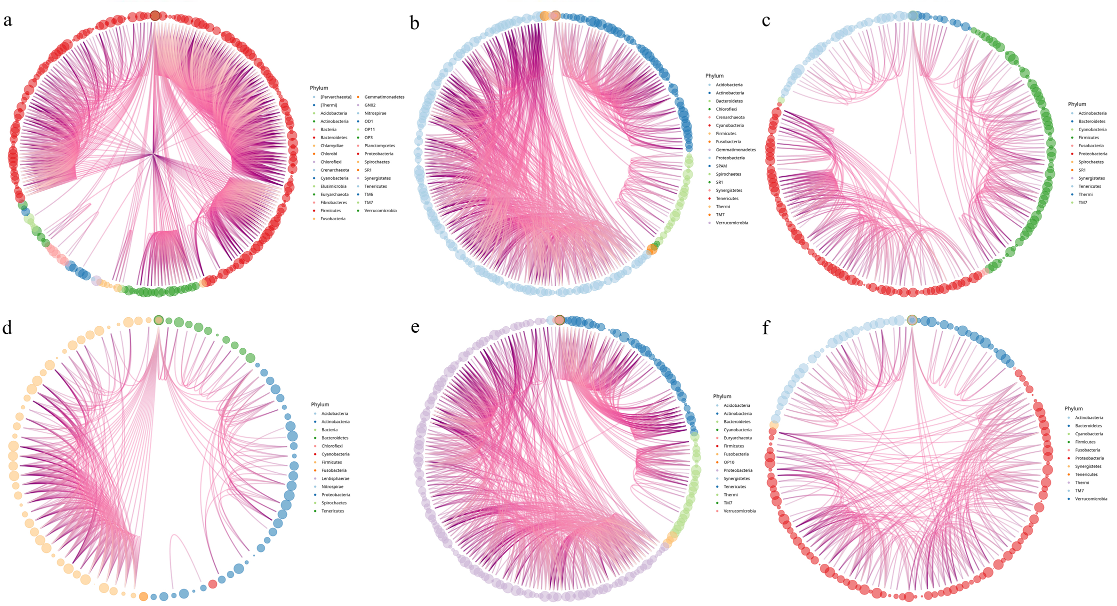

Supplementary Figure 4 The taxonomic distributions of six samples—comprising saliva and stool from three subjects—were analysed using the SparCC method to characterise both the association networks and the phylum-level composition of each sample.

(a–c) Circos plots depicting the patterns of associations within saliva samples from subject A (a), subject M3 (b), and subject F4 (c). (d–f) Circos plots illustrating the association patterns within stool samples from subject A (d), subject M3 (e), and subject F4 (f). In these plots, the circles along the periphery represent individual taxa, with taxa belonging to the same phylum coloured identically. Thin bands connecting operational taxonomic units (OTUs) highlight high-resolution interactions.
